# Impact of community health workers on improving identification and primary care of hypertension among the urban poor – findings from Chhattisgarh state of India

**DOI:** 10.1186/s12875-023-02231-1

**Published:** 2023-12-13

**Authors:** Samir Garg, Mukesh Dewangan, Prabodh Nanda, Ashu Sahu, Lalita Xalxo, Kirtti Kumar Bebarta, Vishnu Gupta, Mohammad Jawed Quereishi, Anand Kumar Sahu, Pradeep Tandan

**Affiliations:** 1 State Health Resource Centre, Chhattisgarh, Raipur India; 2 State Programme Management Unit, National Health Mission, Chhattisgarh, Raipur India

## Abstract

**Background:**

Hypertension is a major health problem globally and in India. Around 60% of people with hypertension in India are not aware that they have the condition. Less than 30% of individuals with hypertension are on treatment. Existing studies have indicated that community health workers (CHWs) can play a useful role in expanding the care for hypertension. Evaluations are needed to study the impact when an intervention is implemented by the government in its existing large-scale CHW programme to improve the identification, regular follow-up and medication adherence for hypertension.

**Methods:**

Chhattisgarh state implemented a pilot intervention to improve screening and follow-up for hypertension by equipping Mitanin-CHWs to measure blood pressure (BP). The study design involved an intervention-group and a comparison-group of urban slum population. The survey covered 5974 individuals (30–79 years age) in intervention-group and 5131 in comparison-group. Multivariate analysis was conducted to find out the effect of intervention on the desired outcomes.

**Results:**

In intervention-group, 80.2% of the individuals (30–79 years age) had been screened for hypertension whereas the proportion was 37.9% in comparison-group. For 47.0% of individuals in intervention-group, Mitanin CHW was the provider who measured BP for the first time. Around 16.3% of individuals in intervention-group and 9.5% in comparison-group had been diagnosed with hypertension. Around 85.9% of hypertension cases in intervention-group and 77.0% in comparison-group were on treatment. BP had been measured in preceding 30 days for 81.8% of hypertension-cases in intervention-group and 64.3% in comparison-group. Around 70.3% of hypertension-cases in intervention-group and 55.1% in comparison-group had taken their complete medication for last seven days. Multivariate analysis showed that CHW intervention was associated significantly with improvements in all the desired outcomes.

**Conclusion:**

Equipping the CHWs to measure BP was effective in increasing the screening and identification of hypertension, regular measurement of BP of individuals with hypertension and the adherence to medication. This shows the potential if the one-million strong work-force of Accredited-Social-Health-Activists (ASHA) CHWs in India gets equipped for this role. Governments need to provide a stronger policy push to get this materialised.

**Supplementary Information:**

The online version contains supplementary material available at 10.1186/s12875-023-02231-1.

## Background

The noncommunicable diseases (NCDs) are a leading cause of ill health and mortality across the world [1, 2]. NCDs currently contribute to 74% of mortality globally [1]. NCDs account for 17 million premature deaths (below age of 70 years) and 84% of those occur in the Low- and Middle-Income Countries (LMICs) including India [1]. In India, NCDs cause an estimated 6 million deaths annually and they constitute two-thirds of all deaths in the country [2].

Among the NCDs, hypertension or high blood pressure is the most prominent condition in terms of prevalence in population [3]. Uncontrolled hypertension causes many severe complications. It damages heart resulting in angina, heart attack, heart failure and irregular heart-beat. Uncontrolled hypertension is the main cause of stroke [3]. Uncontrolled hypertension also damages kidney resulting in kidney failure [3–5].

According to global estimates for 2023, around 1.28 billion people aged 30–79 years have hypertension and two-third of them live in the LMICs [3]. The global prevalence of hypertension has doubled between 1990 and 2019 and the LMICs including India and China accounted for a big share of this increase [3, 6, 7]. The prevalence of hypertension among the population aged 30–79 years was 31.1% in India, close to the global average of 33.1% [7]. In India, around 220 million individuals are estimated to have hypertension [8].

At the same time, the awareness in India on Hypertension has also found to be low [9]. A 2019 review estimated that 58.3% of women and 68.3% of men with hypertension in India were unaware that they have the condition [6]. Only 35.1% of women and 25.1% of men with hypertension in India are under treatment [6]. According to a World Health Organisation (WHO) estimate, only 12% of the persons with hypertension in India have it under control in comparison to the global average of 21% [3, 8].

Globally, studies have indicated that CHWs can play an important role in expanding the effective utilisation of NCD care [10–15]. India has a fifteen years old nationwide programme with around a million CHWs called ASHAs (Accredited Social Health Activists) [16]. The ASHA CHWs have been known for their impactful work on reproductive and child health [16]. However, not much is known about their work on NCDs including hypertension.

A few intervention studies on training ASHAs to work on hypertension have emerged in recent years. A study showed that ASHAs were able to demonstrate substantial knowledge on community counselling for hypertension when a training was provided to them on those actions [17]. An intervention study focused on training ASHAs to deliver advice and measure BP by conducting group meetings of known cases of hypertension found that ASHAs were able to conduct the meetings and the intervention was effective in achieving better BP control [18, 19]. An intervention study involving ASHAs found follow-up through them was effective in better BP control among the known cases of stroke [20]. Another intervention study equipped ASHAs in BP measurement and to link the individual showing high BP with telemedicine using mobile phones. This study did not have a comparison group but found that ASHAs played a positive role in identification of new cases and better BP control among the known cases of hypertension [21]. Another study equipped ASHAs in BP measurement and to link the individuals showing high BP with telemedicine using portable computer tablets. This study did not have a comparison group but showed that the intervention was effective in improving BP control [22].

All of the existing Indian studies have examined the impact of CHWs involving small-scale interventions on hypertension through civil society organisations or the private sector. Evaluations are needed to study the impact when the intervention gets implemented by the government through its existing CHW programme. The present study was aimed at filling this gap.

In Chhattisgarh state of India, there are 71,000 CHWs known as Mitanins and they have proven their effectiveness in the domains of reproductive and child health as well as the communicable diseases [23–28]. They are also considered as a part of the national programme umbrella of ASHA. In urban areas of Chhattisgarh, Mitanin CHWs mainly cover the population living in the slums. The average population looked after by a Mitanin CHW in urban areas is around 700 [23].

In 2019, Chhattisgarh started a pilot intervention for improving screening and primary care of hypertension by involving CHWs [29]. The intervention involved equipping a group of Mitanin CHWs with digital BP monitors and training them on screening and follow-up of hypertension among the urban poor. The main components of the intervention were:

- Each CHW in the intervention area was expected to screen all individuals in 30–79 years age group by measuring their BP once a year.
- Any individual with BP greater than 140/90 mm Hg was to be referred to a medical doctor for confirmation.
- Any individual confirmed for hypertension was to be followed up at home by CHWs through a monthly home visit. The home visit included advice on regular consumption of the medicines prescribed by doctors, measurement of BP and referral if the BP was greater than 140/90 mm Hg.

The present study was aimed at evaluating the above intervention. The specific objectives were to examine the impact of this intervention on the following indicators:

- Screening rate: the proportion of target population (30–79 years age) that could get screened.
- Detection rate: the proportion of individuals with hypertension diagnosed by a medical doctor.
- Treatment rate: the proportion of hypertension cases who were receiving treatment at the time of the survey.
- Follow-up rate: the proportion of hypertension cases who got their BP measured monthly.
- Medication adherence rate: the proportion of hypertension cases who took their medication regularly.

## Methods and materials

Study setting: Chhattisgarh is one of the poorer states in India. Around 25% of the population lives in urban areas and around 40% of the urban population lives in slums [23]. The study was conducted in Raipur, an urban area in Chhattisgarh state. There were 1040 Mitanin CHWs covering around 700,000 population of the urban poor in the Raipur city. At the time of data collection of this study, 500 out of the 1040 CHWs in the city had received training on BP measurement and they had gained experience of one year in that activity. They used digital BP monitors with specified accuracy of ± 5 mm Hg and life of 10,000 readings. The rest 540 CHWs were not equipped to measure BP at that time.

Study design and sampling: The study design involved an intervention group and a comparison group of urban slum population. The intervention group consisted of population covered by CHWs trained to measure BP for a year. The comparison group was also covered by CHWs but without any training or equipment to measure BP.

Since objectives of the study also included assessing the regularity of follow-up and medication adherence among the hypertension cases, the minimum sample size was calculated to yield enough number of hypertension cases in each group. For a confidence level of 95% and desired precision of 5%, a minimum requirement of 384 hypertension cases was calculated for each group. Assuming an average prevalence of 10% in the age 30–79 years, a minimum of 3840 individuals were to be covered in each group.

In the intervention group, 50 CHWs were selected from the list of the 500 CHWs trained on BP measurement. Systematic random sampling was used for the selection. Similarly, 50 CHWs were selected from the list of 540 CHWs not trained on BP measurement. All individuals in 30–79 years age residing in the designated work-area of the above CHWs were to be covered. The survey was able to cover 5974 individuals of that age in the intervention group and 5131 in the comparison group.

Data collection: A structured questionnaire was used to collect data on socio-demographic characteristics of individuals and the status of screening and diagnosis for hypertension (Additional File S1). For those reporting themselves as diagnosed cases of hypertension, the information was confirmed by the surveyors by examining the medicines they had taken, prescriptions or any other available medical records or asking detailed questions on where they were diagnosed and what was the BP reading. For all confirmed cases of hypertension, further questions were asked on whether they were on treatment, regularity of BP measurement and regularity in taking medication. The survey questionnaire was field tested. The data collection was carried out in January 2021.

Data analysis: The list of study variables is given in Additional File S2. High BP was defined as anyone with a systolic BP above 140 mm mm Hg or diastolic BP above 90 mm Hg [30]. Follow-up for BP measurement was defined as regular if a hypertension case had BP measured over the past one month (30 days). Medication was considered as regular if the individual consumed hypertension medicines on all seven days of the preceding week. The recall period for consuming medication was kept as one week because the recall for a longer duration was found to be unreliable during the field testing of the draft tool.

Descriptive analysis was done using cross-tabulations. Test of significance at 95% confidence were reported while comparing the important outcome indicators for intervention and comparison groups. Multivariate analysis was conducted to find out the effect of intervention on the desired outcomes i.e., screening for hypertension, identification of hypertension cases, hypertension cases being under treatment, monthly measurement of BP for follow-up and regular consumption of medicines for hypertension. The data analysis was done using STATA 5 software.

## Results

The intervention group as well as the non-intervention group were from the urban slum population of Raipur district of Chhattisgarh. The socio-demographic profile of the sample is given in Table 1.

**Table 1:** Socio-demographic profile of the sample

Variable	Category	All (%)	Intervention group (%)	Comparison group (%)	Test of significance (*p* value)
		n = 11,105	n = 5974	n = 5131	
Caste	Scheduled Tribes	1332 (12.0)	596 (10.0)	736 (14.3)	< 0.01
	Scheduled castes	2664 (24.0)	1164 (19.5)	1500 (29.2)	< 0.01
	Other backward classes	5456 (49.1)	3260 (54.6)	2196 (42.8)	< 0.01
	Others	1653 (14.9)	954 (16.0)	699 (13.6)	< 0.01
Sex	Male	5469 (49.3)	2873 (48.1)	2596 (50.6)	< 0.01
	Female	5636 (50.7)	3101 (51.9)	2535 (49.4)	< 0.01
Education	Not Literate	4004 (36.1)	1931 (32.3)	2073 (40.4)	< 0.01
	Primary	3115 (28.0)	1635 (27.4)	1480 (28.8)	0.10
	Secondary	2632 (23.7)	1525 (25.5)	1107 (21.6)	< 0.01
	Above secondary	1354 (12.2)	833 (14.8)	471 (9.2)	< 0.01
Mean age (years)		46.0	46.8	45.1	< 0.01
Mean family size		5.3	5.4	5.1	< 0.01
Mean distance from nearest health facility (kilometres)		1.8	1.5	2.2	< 0.01

### Screening for hypertension

**Fig. 1:**
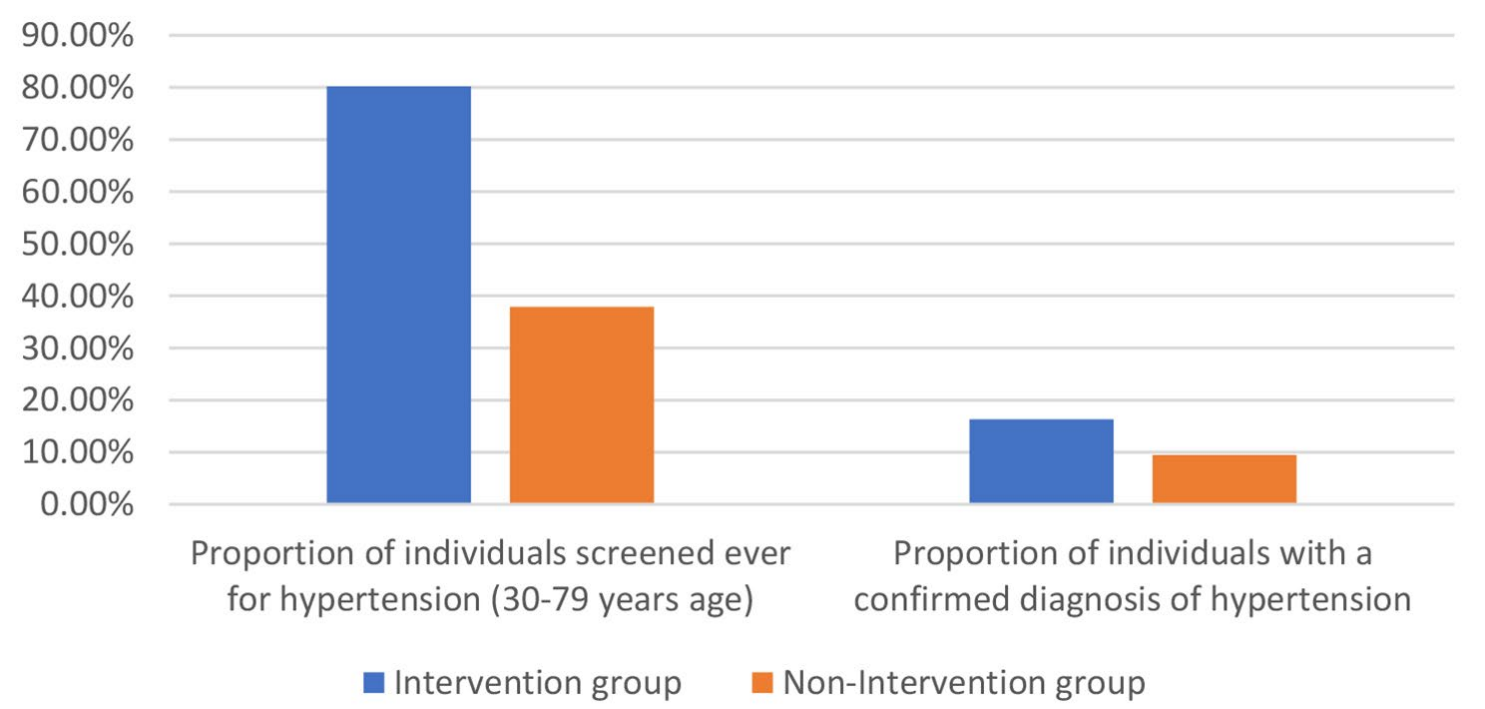
Screening and detection of hypertension in intervention (n = 5974) and non-intervention (n = 5131) groups

The proportion of individuals (aged 30–79 years) who had been screened at least once for hypertension in their life time was 80.2% in the intervention group and 37.9% in the non-intervention group (*p* < 0.01) (Fig. 1).

Table 2 provides the information on various health providers utilized when the individuals got screened for hypertension for the first time in their life. This indicates the impact of CHWs on screening as the first ever measurement of BP for 47% of individuals in the intervention group was done by Mitanin CHWs.

**Table 2:** Type of provider who measured BP first time for the individuals (30–79 years age)

Category	All (%) n = 11,105	Intervention group (%) n = 5974	Non-Intervention group (%) n = 5131	Test of significance (*p* value)
Government facility	1026 (9.2)	554 (9.3)	472 (9.2)	0.86
Private facility	2906 (26.2)	1433 (24.0)	1473 (28.7)	< 0.01
Mitanin CHW	2806 (25.3)	2806 (47.0)	0 (0.0)	< 0.01
BP never measured	4367 (39.3)	1181 (19.8)	3186 (62.1)	< 0.01

#### Identification of hypertension

The proportion of individuals with a confirmed diagnosis of hypertension i.e., the rate of identification was 16.3% for the intervention group and 9.5% for the non-intervention group (*p* < 0.01) (Fig. 1).

#### Proportion of hypertension cases under treatment

**Fig. 2:**
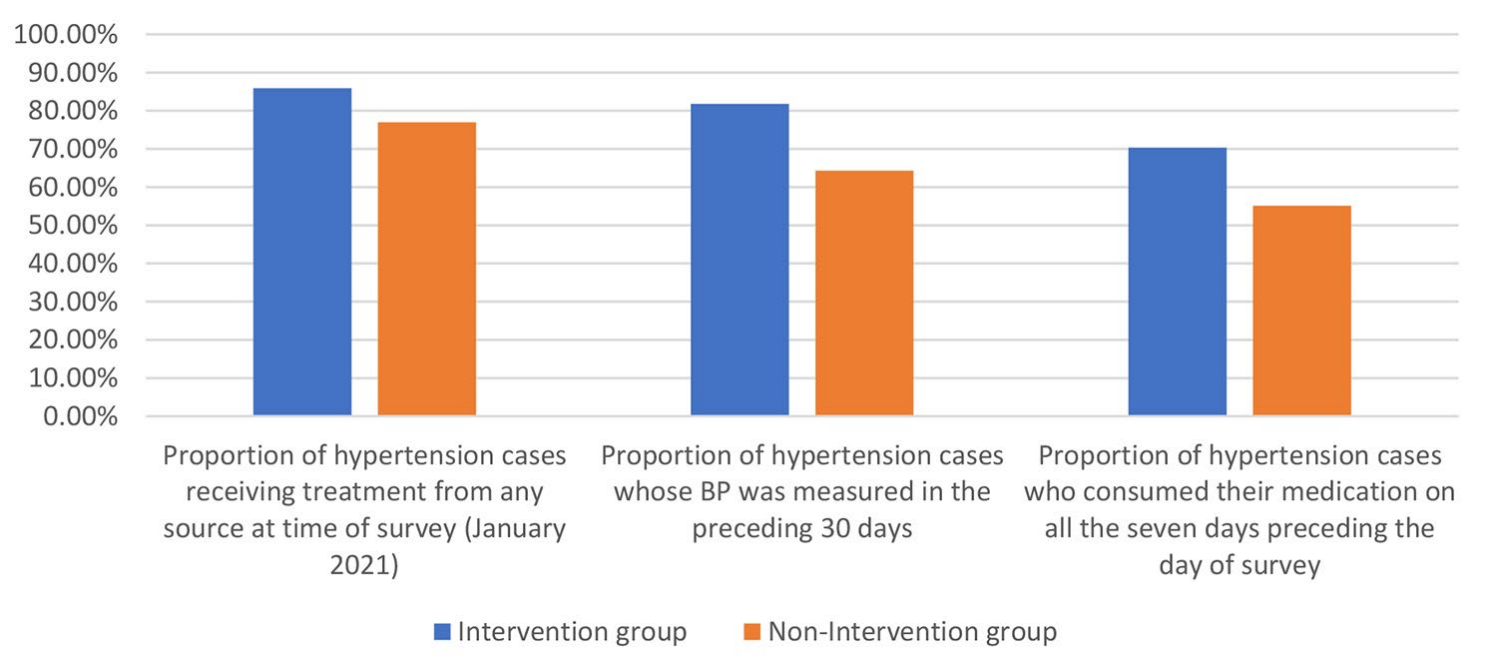
Indicators of treatment and regular follow-up of hypertension cases in the intervention (n = 978) and non-intervention (n = 488) groups

The proportion of hypertension cases who were receiving treatment at the time of the survey was 85.9% in the intervention group and 77.0% in the non-intervention group (*p* < 0.01) (Fig. 2).

#### Regular BP measurement for follow-up

The proportion of hypertension cases whose BP was measured in the preceding 30 days was 81.8% in the intervention group and 64.3% in the non-intervention group (*p* < 0.01) (Fig. 2).

#### Medication adherence

The proportion of hypertension cases who consumed their medication on all the seven days preceding the day of survey was 70.3% in the intervention group and 55.1% in the non-intervention group (*p* < 0.01) (Fig. 2).

### Results of multivariate analysis

The results of logistic regression model to find out the determinants of being screened for hypertension are given in Table 3. It shows that an individual in the intervention group was significantly more likely to get screened for hypertension than the non-intervention group. The chances of getting screened were also higher for women, the elderly and the those with higher education (Table 3).

**Table 3:** Logistic regression model to find out the impact of CHW intervention on screening for hypertension

Outcome variable: Ever screened for hypertension (Yes/No)						
Independent variables: Intervention, Age, Sex, Caste, Education, Family size, Distance from nearest health facility						
Model: Logistic regression					N = 11,105	
Variable	Adjusted Odds Ratio	Std. Err.	z	P > z	95% Confidence Interval	
**Intervention**	Ref (Comparison group)					
Intervention group	7.01	0.33	41.08	< 0.01	6.39	7.69
**Age**	1.03	0.00	15.37	< 0.01	1.03	1.04
**Sex**	Ref (Male)					
Female	1.97	0.09	14.45	< 0.01	1.79	2.15
**Caste**	Ref (Scheduled Tribes)					
Scheduled Castes	0.92	0.07	-1.03	0.30	0.79	1.07
Other Backward Classes	0.89	0.06	-1.64	0.10	0.77	1.02
Others	0.79	0.07	-2.74	0.01	0.66	0.93
**Education**	Ref (Not Literate)					
Primary	1.01	0.06	0.17	0.87	0.90	1.13
Secondary	1.14	0.07	2.02	0.04	1.00	1.29
Above secondary	1.37	0.11	3.89	< 0.01	1.17	1.60
**Family size**	1.00	0.01	-0.02	0.98	0.98	1.02
**Distance from nearest facility**	1.06	0.02	2.76	0.01	1.02	1.10

An individual in the intervention group was more likely to get diagnosed with hypertension than the non-intervention group (Table 4). The probability of getting diagnosed with hypertension also depended on age, sex, caste and education of the individual (Table 4).

**Table 4:** Logistic regression model to find out the impact of CHW intervention on diagnosis of hypertension

Outcome variable: Ever diagnosed (confirmed) as hypertensive (Yes/No)						
Independent variables: Intervention, Age, Sex, Caste, Education, Family size, Distance from nearest health facility						
Model: Logistic regression					N = 11,105	
Variable	Adjusted Odds Ratio	Std. Err.	z	P > z	95% Confidence Interval	
**Intervention**	Ref (Comparison group)					
Intervention group	1.59	0.11	6.93	< 0.01	1.40	1.82
**Age**	1.08	0.00	29.63	< 0.01	1.07	1.09
**Sex**	Ref (Male)					
Female	2.24	0.15	12.01	< 0.01	1.96	2.55
**Caste**	Ref (Scheduled Tribes)					
Scheduled Castes	1.04	0.12	0.34	0.74	0.83	1.29
Other Backward Classes	1.04	0.11	0.42	0.67	0.85	1.28
Others	1.30	0.16	2.20	0.03	1.03	1.64
**Education**	Ref (Not Literate)					
Primary	1.04	0.08	0.52	0.60	0.89	1.22
Secondary	1.17	0.11	1.70	0.09	0.98	1.39
Above secondary	1.22	0.14	1.77	0.08	0.98	1.53
**Family size**	1.02	0.01	1.73	0.08	1.00	1.04
**Distance from nearest facility**	0.94	0.03	-1.75	0.08	0.88	1.01

Table 5 shows the results of multivariate analysis to find out the determinants of hypertension cases being on treatment. The hypertension cases in intervention group were significantly more likely to be on treatment than the non-intervention group. The elderly and women cases were more likely to be on treatment (Table 5).

**Table 5:** Logistic regression model to find out the impact of CHW intervention on hypertension cases being on treatment

Outcome variable: Whether the hypertension case was receiving treatment for it (Yes/No)						
Independent variables: Intervention, Age, Sex, Caste, Education, Family size, Distance from nearest health facility						
Model: Logistic regression					N = 1464	
Variable	Adjusted Odds Ratio	Std. Err.	z	P > z	95% Confidence Interval	
**Intervention**	Ref (Comparison group)					
Intervention group	1.77	0.13	7.80	< 0.01	1.53	2.04
**Age**	1.08	0.00	27.25	< 0.01	1.07	1.08
**Sex**	Ref (Male)					
Female	2.15	0.15	10.66	< 0.01	1.87	2.48
**Caste**	Ref (Scheduled Tribes)					
Scheduled Castes	0.97	0.12	-0.23	0.82	0.77	1.23
Other Backward Classes	1.02	0.11	0.21	0.84	0.82	1.27
Others	1.31	0.17	2.11	0.03	1.02	1.68
**Education**	Ref (Not Literate)					
Primary	1.07	0.09	0.79	0.43	0.90	1.27
Secondary	1.21	0.12	1.94	0.05	1.00	1.46
Above secondary	1.22	0.15	1.64	0.10	0.96	1.56
**Family size**	1.02	0.01	1.58	0.11	1.00	1.04
**Distance from nearest facility**	0.95	0.04	-1.44	0.15	0.88	1.02

The likelihood of the BP measurement in last 30 days was greater for the hypertension cases in the intervention group (Table 6). The elderly and women cases were more likely to get their BP checked (Table 6).

**Table 6:** Logistic regression model to find out the impact of CHW intervention on regular blood pressure measurement for follow-up

Outcome variable: BP measured in preceding 30 days (Yes/No)						
Independent variables: Intervention, Age, Sex, Caste, Education, Family size, Distance from nearest health facility						
Model: Logistic regression					N = 1464	
Variable	Adjusted Odds Ratio	Std. Err.	z	P > z	95% Confidence Interval	
**Intervention**	Ref (Comparison group)					
Intervention group	2.10	0.16	9.64	< 0.01	1.81	2.45
**Age**	1.08	0.00	26.16	< 0.01	1.07	1.08
**Sex**	Ref (Male)					
Female	2.13	0.16	10.15	< 0.01	1.84	2.47
**Caste**	Ref (Scheduled Tribes)					
Scheduled Castes	0.96	0.12	-0.31	0.76	0.75	1.23
Other Backward Classes	0.92	0.10	-0.76	0.45	0.74	1.14
Others	1.22	0.16	1.50	0.13	0.94	1.57
**Education**	Ref (Not Literate)					
Primary	1.13	0.10	1.36	0.17	0.95	1.34
Secondary	1.22	0.12	1.96	0.05	1.00	1.49
Above secondary	1.19	0.15	1.33	0.18	0.92	1.53
**Family size**	1.02	0.01	1.85	0.06	1.00	1.04
**Distance from nearest facility**	0.97	0.04	-0.90	0.37	0.90	1.04

The likelihood of medication adherence (consuming medicines on all the seven preceding days) was significantly greater for the hypertension cases in intervention group than the non-intervention group. The above indicator was also likely to be better for the elderly and the women (Table 7).

**Table 7:** Logistic regression model to find out the impact of CHW intervention on taking hypertension medication regularly

Outcome variable: Medication consumed on all preceding seven days (Yes/No)						
Independent variables: Intervention, Age, Sex, Caste, Education, Family size, Distance from nearest health facility						
Model: Logistic regression					N = 1464	
Variable	Adjusted Odds Ratio	Std. Err.	z	P > z	95% Confidence Interval	
**Intervention**	Ref (Comparison group)					
Intervention group	2.09	0.17	9.06	< 0.01	1.78	2.45
**Age**	1.08	0.00	25.19	< 0.01	1.07	1.08
**Sex**	Ref (Male)					
Female	2.04	0.16	8.95	< 0.01	1.74	2.38
**Caste**	Ref (Scheduled Tribes)					
Scheduled Castes	0.99	0.13	-0.10	0.92	0.75	1.29
Other Backward Classes	1.06	0.13	0.48	0.63	0.83	1.35
Others	1.35	0.19	2.14	0.03	1.03	1.79
**Education**	Ref (Not Literate)					
Primary	1.08	0.10	0.82	0.42	0.90	1.30
Secondary	1.15	0.13	1.31	0.19	0.93	1.43
Above secondary	1.21	0.16	1.39	0.16	0.93	1.58
**Family size**	1.01	0.01	0.85	0.40	0.99	1.04
**Distance from nearest facility**	1.01	0.03	0.41	0.68	0.96	1.06

## Discussion

The current study reports the impact of CHWs on detection and management of hypertension at the population level when the intervention was made in the context of an existing large-scale government programme. It showed that the intervention had substantial positive impact on the desired outcomes. This finding is in line with what the existing global literature indicates [10, 11].

In terms of screening for hypertension, our study found that 80.2% of population coverage could be achieved through CHWs, within a year of the intervention. This is significant because as per existing estimates of WHO, nearly 60% of those with hypertension in India are unaware of their status [8]. This was confirmed in the current study where only 38% of the individuals in the non-intervention group reported ever being screened for hypertension. This reflected in the lower detection in non-intervention group with 9.5% of individuals confirmed for hypertension, whereas the proportion in the intervention group was 16.3%. An earlier study had reported the prevalence of hypertension as 23.6% in this age group and the WHO estimate for India is of 31.1% [8, 21]. What explains the lower prevalence reported in the current study? Around 20% of the target population was yet to be screened in the intervention group. But more importantly, many individuals showing high BP during screening might not have accessed the services necessary for confirmation of hypertension. This shows the need for additional interventions to improve the access of urban poor to clinical services on confirmation of hypertension and initiating treatment. In this regard, the recent move of Indian government to set up public clinics at every 15,000 population in urban areas has potential to be a game changer in the coming years [31].

In the present study, The CHW intervention also helped in increasing the proportion under treatment among the confirmed cases of hypertension. In the intervention group, 85.9% of hypertension cases were found to be under treatment. An earlier study had reported 77.6% as under treatment where CHWs intervened [22].

Regular measurement of BP is an essential part of the primary care for hypertension. Earlier Indian studies have not reported on this indicator in the context of a CHW-based intervention. In the present study, 81.8% of hypertension cases in the intervention area had their BP measured in the preceding 30 days. The CHWs played a key role by measuring their BP regularly.

Adherence to medication i.e., taking the medication daily is a very significant objective in the primary care of many chronic diseases including hypertension. Earlier Indian studies on CHWs have not covered this aspect. In the intervention group of current study, 70.3% of hypertension cases took their medication on all the preceding seven days. The CHW intervention was significantly associated with better rate of medication adherence.

The intervention studied here was implemented through the existing government mechanisms. Many innovative programmes initiated by non-government agencies face challenges in remaining in operation for long periods of time as they have to depend upon short-term or uncertain funding. In the intervention studied here, the initiative was to integrate the work on hypertension control with the existing ASHA programme instead of creating a stand-alone programme on hypertension. This had advantages in terms of stable government funding and support. E.g., the battery replacement costs got covered through the existing untied funds that government provides. An important aspect to keep in mind though will be the amount of time ASHAs can spare for such work in addition to their existing duties. The size of the population each ASHA looks after will be a key factor in this regard. There may also be a need to rationalise the time use of ASHAs by reducing some of the work burden related to relatively less useful tasks. Another important aspect requiring attention will be the arrangements for periodic identification and replacement of the out of order BP monitors.

In recent years, India has stepped up its efforts to control NCDs including hypertension. National guidelines on NCD care have been implemented and annual population-based screening of individuals above age of 30 years has been recommended for timely detection of hypertension [30]. All government hospitals and primary health centres are being equipped to diagnose hypertension and initiate treatment [30]. In addition, India launched a nationwide initiative called Comprehensive Primary Health Care (CPHC) in 2018 [32]. Under CPHC, clinics known as Health and Wellness Centres (HWCs) have been established at a rural population of 5000 to carry out population-based screening and provide the primary care services for a wide range of health needs including NCDs such as hypertension [32]. With the emergence of the Health and Wellness Centres (HWCs), ASHA CHWs are being considered a part of the frontline team providing primary care services [32].

The WHO has recognised the ASHA CHWs as global leaders in health for the role played by them in providing services during the COVID-19 pandemic [33]. Apart from the public health functions and vaccination for COVID-19, they helped in maintaining the essential health services ranging from immunisation, ante-natal care and malaria control. In addition, they became a vehicle for reaching to the patients at their homes to ensure continuity of treatment including for chronic diseases such as hypertension and diabetes-mellitus [34]. The study period of present study covered the first wave of COVID-19 in India [35]. The high coverage of population achieved by CHWs in Chhattisgarh during that period is further remarkable because they were able to achieve it during the challenging times of the pandemic. It could also be the case that more people utilized the services of CHWs during of the pandemic because of their constrained access to the other providers. This also highlights the importance of equipping CHWs in primary care of hypertension to better prepare the health systems to sustain essential services for NCDs during emergencies.

Screening a large population and following up all the hypertension cases is a herculean task for any facility. The individuals above age of 30 years constitute around 37% of population in India [30]. This means that a health centre at 5000 population has to screen around 1850 individuals annually. If identified properly, around 500 of them would be expected to be confirmed as hypertensive [30]. The confirmed cases of hypertension would require monthly measurement of BP. A heath centre with a staffing of two to three health professionals can get overwhelmed by the workload related to hypertension alone. The availability of CHWs in the Indian primary care system offers the potential to tackle this burden.

Apart from the large numbers, ensuring adherence to medication for hypertension indicates the need for services not just at the facilities, but also at the home level. CHWs are an arm of the primary care system that can reach to the home level for a wide range of interventions, including for NCDs. India has a huge gap in clinic visits for hypertension and there is a shortage of clinical services for hypertension [9, 36, 37]. India has a large presence of private sector in healthcare but the private facilities have shown little interest in population-based screening, especially in covering the vulnerable communities [38]. The private hospitals and clinics face serious challenges in ensuring regular clinic visits of patients, resulting in insufficient adherence to medication and poor control of hypertension [39]. The ASHA CHWs thus can be a suitable vehicle for expanding population based screening and regular follow-up for hypertension. ASHAs belong to and live in the communities that they serve and they enjoy their trust [16]. This puts them at an advantage in providing counselling more effectively for a variety of health issues including the NCDs [16]. The close to community availability of ASHAs makes it easier for patients to access their services [16]. Their proximity can also help in reducing the money and time spent by patients. The access to care for NCDs is highly inequitable in India and CHWs can help in mitigating the existing disparities to a great extent.

Researchers have recommended a greater involvement of CHWs in NCD work to narrow down the access gap in India [37]. The current study provides further evidence in support of that recommendation. However, several steps may be necessary to implement this recommendation effectively. In 2018, a training module was designed at the national level to train the ASHA CHWs on NCDs including hypertension [40]. It included a section on the skill of BP measurement but, the decision on equipping the CHWs in BP measurement was left to the states [40]. Most of the states are yet to take a clear decision in this regard. Chhattisgarh has been one of the pioneer states in starting the initiative to equip the CHWs in BP measurement. Starting from 2019, the state equipped 3700 CHWs in urban areas and 27,000 CHWs in rural areas by March 2023 [41]. Apart from providing the training and equipment, paying the CHWs is another important aspect. In India, ASHA CHWs do not earn a monthly salary and task-based incentives constitute the main mode of payment for them [23]. A cash incentive has been introduced by the national government that allows the ASHA CHWs to earn a small amount every time they help in identification of a hypertension case and when their home visits ensure regular clinic visits by hypertension cases [42]. The current study shows that if CHWs get equipped for BP measurement, the identified cases of hypertension rise substantially. Thus, measuring BP can help the ASHA CHWs to earn better as well.

The current study recommends a stronger push by the national and state governments to equip all the one million ASHAs in the country to measure BP. This will require an increase in the public funding. In Chhattisgarh, supportive supervision has been recognised as a key strength behind its successful CHW-based interventions [23–28]. This factor could have helped the efforts of Chhattisgarh on the hypertension front as well. This suggests that strengthening the supportive supervision in the ASHA CHW programme should be a priority across the country. A CHW-based intervention cannot succeed in isolation, especially in the context of diseases such as hypertension that require care from clinicians also. In this regard, the newly established network of 150,000 health and wellness centres, each covering a population of around 5000, can play a crucial role. Equipping the CHWs in BP measurement, engaging them in primary care of hypertension in tandem with increasing their remuneration and integrating them with the primary care network of health and wellness centres will be the measures vital for achieving the desired population coverage in India.

Further research is recommended to identify the factors that facilitated the CHWs in Chhattisgarh in being effective in their role in screening and primary care of hypertension. Further research is recommended to find ways to scale up such initiatives, especially in the LMIC contexts. Studies are needed to examine if similar interventions for other important NCDs, type-2 diabetes for instance, can be feasible through CHWs.

Limitations: The survey in current study did not measure BP of the participants and therefore it did not find out if BP was controlled better with the CHW intervention. Modifications in lifestyle and diet are also important components of primary care of hypertension but the current study did not focus on these aspects [43].

## Conclusion

The intervention of equipping the CHWs to measure BP was effective in increasing the screening and identification of hypertension, regular measurement of BP of the identified hypertension cases and their adherence to medication. Remarkable improvements in primary care of hypertension were achieved in the context of a government funded and large-scale CHW programme. This shows the potential that can be realized by training the one million strong work-force of ASHA CHWs in India. Governments in India and other LMICs need to provide a stronger policy push to get this materialised if the existing disparities in access to NCD care are to be addressed and the desired population coverage is to be achieved.

### Electronic supplementary material

Below is the link to the electronic supplementary material.

Supplementary Material 1

Supplementary Material 2

## Data Availability

The datasets used and/or analysed during the current study are available from the corresponding author and State Health Resource Centre, Chhattisgarh on reasonable request.

## Abbreviations

ASHA: Accredited Social Health Activist
BP: Blood Pressure
CHWs: Community Health Workers
CI: Confidence Interval
Hg: Mercury
LMICs: Low- and middle-income countries
mm: Millimetre
NCD: Non-Communicable Diseases
WHO: World Health Organisation
