# Supplementary material for: Impact of community health workers on improving identification and primary care of hypertension among the urban poor – findings from Chhattisgarh state of India
Source: BMC Prim Care. 2023 Dec 13;24:272. doi: 10.1186/s12875-023-02231-1 (PMC10717607; doi:10.1186/s12875-023-02231-1)
Supplement: Supplementary file 1 — Supplementary Material 1 [file 12875_2023_2231_MOESM1_ESM.pdf]

Study on primary care for hypertension in urban poor population
Household survey questionnaire and guide

Information Sheet

Your house has been chosen to take part in the study on primary care for hypertension in urban poor population, being conducted by the State Health Resource Centre, Chhattisgarh. This research aims to inform us about the screening and treatment for hypertension among the population in age of 30 to 79 years. Our interviewer will ask questions about you and your family. Questions are about household, individuals, screening for hypertension, treatment of individuals diagnosed with hypertension and their follow-up. The questions will take about 20 to 30 minutes. We may contact you to check the work of your interviewer. We may contact you again for further information. We are bound by guidelines to use your information for statistical research only and to keep it confidential. The guidelines prohibit us from giving anyone any information that may identify you or your family without your consent. You have all liberty to either participate or withdraw from the study at any point of time.

Informed Consent:

(To be obtained from each individual participating in the survey)

Declaration (To be obtained from each individual participating in the survey)

I (Title)......(Name) ____ declare that I have been informed regarding the nature of the study in my own language and I am voluntarily agreeing to participate in the study

Signature of the Participant (or the legal representative in case of an illiterate respondent):____

Date: _________________

Place: _________________

Interviewer Name: ___________________________

Interviewer Signature: _________________________

Section-A

Descriptive household information

(Please fill for each household in the sample urban slum allocated to you)

A1. City ___________________ ID ________

A2. Ward No._______________ID __________

A3. Name of urban slum______________ID______

A4. Household ID _____________

A5. Surveyor's Name ______________ID ______________

A6. Date of Survey (dd/mm/yy) _________________

A7. Name of the Household head _________________Sex________________Age ____________

A8. Name od respondent answering Section A_________________Sex________________Age ____________

A9. Distance of nearest public health facility (in km)   ________________________

A10. Social group (caste) of the household

i) Scheduled Tribes (ST)

ii) Scheduled Castes (SC)

iii) Other Backward Classes (OBC)

iv) Others

A11. No. of members in the household____________________________

(Please list all members of the household below, starting from the oldest individual and then in descending order of age)

Sl.	Name of household member	Sex (Male -1, Female -2, Other -3)	Age (in no. of years completed)

Section-B

Information on screening of individuals aged 30 to 79 years

(Please ask each individual aged 30 - 79 years identified through the question A10)

Sl.	B1. Name of the individual	B2.Age<br><br>(in no. of years completed)	B3. Sex<br><br>(Male-1, Female-2, Other-3)	B4. Highest education attained<br><br>(Not literate -1, Primary -2, Secondary -3, Above secondary -4)	B5. Has your blood pressure been measured ever?<br>(Yes- 1, No- 2)<br><br>*(Note: If No - please do not ask the remaining questions from the individual, If Yes – go to next question)*	B6. Who had measured your blood pressure for the first time?<br>(Public sector provider other than Mitanin -1, Mitanin - 2, Private sector provider - 3)	B7. Have you ever been diagnosed with high blood pressure disease (hypertension) by a medical doctor?<br>(Yes- 1, No- 2)<br><br>*(Note: If No - please do not ask the remaining questions from the individual, If Yes – go to Section C)*

Section-C

Information on treatment and follow-up of individuals aged 30 to 79 years and diagnosed with hypertension

(Please ask each individual answering yes to the question B7)

Sl. of individual *(please retain the same sl. no. as in section B)*	Name of the individual	C1. Are you currently taking any medical treatment for hypertension? (Yes-1, No-2)	C2. Was your blood pressure measured in the preceding 30 days? (Yes-1, No-2)	C3. For each of the preceding 7 days, did you consume the medication prescribed for hypertension? (starting from yesterday)						
				Day 1 (Yes-1, No-2)	Day 2 (Yes-1, No-2)	Day 3 (Yes-1, No-2)	Day 4 (Yes-1, No-2)	Day 5 (Yes-1, No-2)	Day 6 (Yes-1, No-2)	Day 7 (Yes-1, No-2)
