# Supplementary material for: Impact of community health workers on improving identification and primary care of hypertension among the urban poor – findings from Chhattisgarh state of India
Source: BMC Prim Care. 2023 Dec 13;24:272. doi: 10.1186/s12875-023-02231-1 (PMC10717607; doi:10.1186/s12875-023-02231-1)
Supplement: Supplementary file 2 — Supplementary Material 2 [file 12875_2023_2231_MOESM2_ESM.docx]

**Addition File – Table S1: List of study variables**

| **Variable name** | **Description** | **Type of variable** | **Categories** |
| --- | --- | --- | --- |
| Caste | Social group the individual belongs to | Categorical | Scheduled Tribes |
|  |  |  | Scheduled castes |
|  |  |  | Other backward classes |
|  |  |  | Others |
| Sex | Sex of the individual | Categorical | Male |
|  |  |  | Female |
| Education | Education attained by the individual |  | Not Literate |
|  |  | Categorical | Primary |
|  |  |  | Secondary |
|  |  |  | Above secondary |
| Age | Age of the individual | Continuous |  |
| Family size | Size of the family individual belongs to | Continuous |  |
| Distance from nearest health facility | Distance from nearest health facility | Continuous |  |
| Screened ever | Whether the individual’s BP was measured ever | Binary | Yes/No |
| Type of health provider | The health facility or worker who measured the individual’s BP for the first time | Categorical | Public facility |
|  |  |  | Private facility |
|  |  |  | Mitanin CHW |
|  |  |  | BP never measured |
| Diagnosed | Whether the individual was confirmed with hypertension | Binary | Yes/No |
| Under treatment | Whether the individual was receiving treatment for hypertension from any source at the time of survey | Binary | Yes/No |
| BP measured in preceding 30 days | Whether BP was measured of the individual with hypertension in preceding 30 days | Binary | Yes/No |
| No. of days medication consumed in preceding seven days | No. of days the individual with hypertension consumed medication in the preceding seven days | Continuous |  |
| Medication consumed all the seven preceding days | Whether the individual with hypertension consumed medication on all the preceding seven days | Binary | Yes/No |
